# Climate-Smart Design for Ecosystem Management: A Test Application for Coral Reefs

**DOI:** 10.1007/s00267-016-0774-3

**Published:** 2016-10-12

**Authors:** Jordan M. West, Catherine A. Courtney, Anna T. Hamilton, Britt A. Parker, Susan H. Julius, Jennie Hoffman, Karen H. Koltes, Petra MacGowan

**Affiliations:** 1 Office of Research and Development, U.S. Environmental Protection Agency, 1200 Pennsylvania Ave, NW (8601P), Washington, DC 20460 USA; 2 Tetra Tech, Inc., 737 Bishop St., Suite 2340, Honolulu, HI 96813-3201 USA; 3 Tetra Tech, Inc., Center for Ecological Sciences, 502 W. Cordova Road, Suite C, Santa Fe, NM 87505 USA; 4 The Baldwin Group, Inc., NOAA Coral Reef Conservation Program, SSMC4, N/OCM6, Rm 10329, 1305 East West Hwy, Silver Spring, MD 20910 USA; 5 Private Consultant, 4755 Northeast Lambs Lane, Poulsbo, WA 98370 USA; 6 U.S. Department of the Interior, Office of Insular Affairs, MS 2429, 1849 C St. NW, Washington, DC 20240 USA; 7 The Nature Conservancy, 74 Wall Street, Seattle, WA 98121 USA

**Keywords:** Climate change, Vulnerability, Adaptation planning, Natural resource management, Coral reefs, Decision making

## Abstract

The interactive and cumulative impacts of climate change on natural resources such as coral reefs present numerous challenges for conservation planning and management. Climate change adaptation is complex due to climate-stressor interactions across multiple spatial and temporal scales. This leaves decision makers worldwide faced with local, regional, and global-scale threats to ecosystem processes and services, occurring over time frames that require both near-term and long-term planning. Thus there is a need for structured approaches to adaptation planning that integrate existing methods for vulnerability assessment with design and evaluation of effective adaptation responses. The Corals and Climate Adaptation Planning project of the U.S. Coral Reef Task Force seeks to develop guidance for improving coral reef management through tailored application of a climate-smart approach. This approach is based on principles from a recently-published guide which provides a framework for adopting forward-looking goals, based on assessing vulnerabilities to climate change and applying a structured process to design effective adaptation strategies. Work presented in this paper includes: (1) examination of the climate-smart management cycle as it relates to coral reefs; (2) a compilation of adaptation strategies for coral reefs drawn from a comprehensive review of the literature; (3) in-depth demonstration of climate-smart design for place-based crafting of robust adaptation actions; and (4) feedback from stakeholders on the perceived usefulness of the approach. We conclude with a discussion of lessons-learned on integrating climate-smart design into real-world management planning processes and a call from stakeholders for an “adaptation design tool” that is now under development.

## Introduction

Given increasingly abundant and compelling evidence for climate change impacts on coral reefs as well as many other ecosystems (Hughes et al. 2003; Parmesan and Galbraith 2004; Parmesan and Yohe 2003; Root et al. 2003; Walther 2010), there is wide recognition that natural resource management must integrate climate change impacts into planning processes in order to be effective (Dessai et al. 2009; Hughes et al. 2003). Many managers have begun to consider climate change in developing reef management strategies (Keener et al. 2012; Levy and Ban 2013; Marshall et al. 2009; The Nature Conservancy 2009, 2010). However, the process of developing and implementing meaningful climate change adaptation options can be challenging due to complexities associated with interactions among climate change and other stressors across multiple spatial and temporal scales; near- and long-term manifestation of impacts; complex time horizons associated with management actions (lead times, response times); multiple uses and ecosystem services; and the multiple management contexts within which the conservation planning takes place. This has led to adaptation planning lagging behind consideration of climate change impacts and vulnerability assessments (Johnson and Weaver 2009).

Managers are requesting tools that will help them in this endeavor. One tool is a recently-released Climate-Smart Conservation guide (Stein et al. 2014). Climate-smart planning provides a general approach for adopting “forward-looking goals” that consider natural resource vulnerabilities to climate change and a guided process to develop and implement strategies crafted to address those vulnerabilities. Using illustrative steps similar to any management planning approach (Conservation Measures Partnership 2013), the climate-smart planning cycle explicitly incorporates principles that are responsive to the challenges of climate change adaptation.

**Fig. 1:**
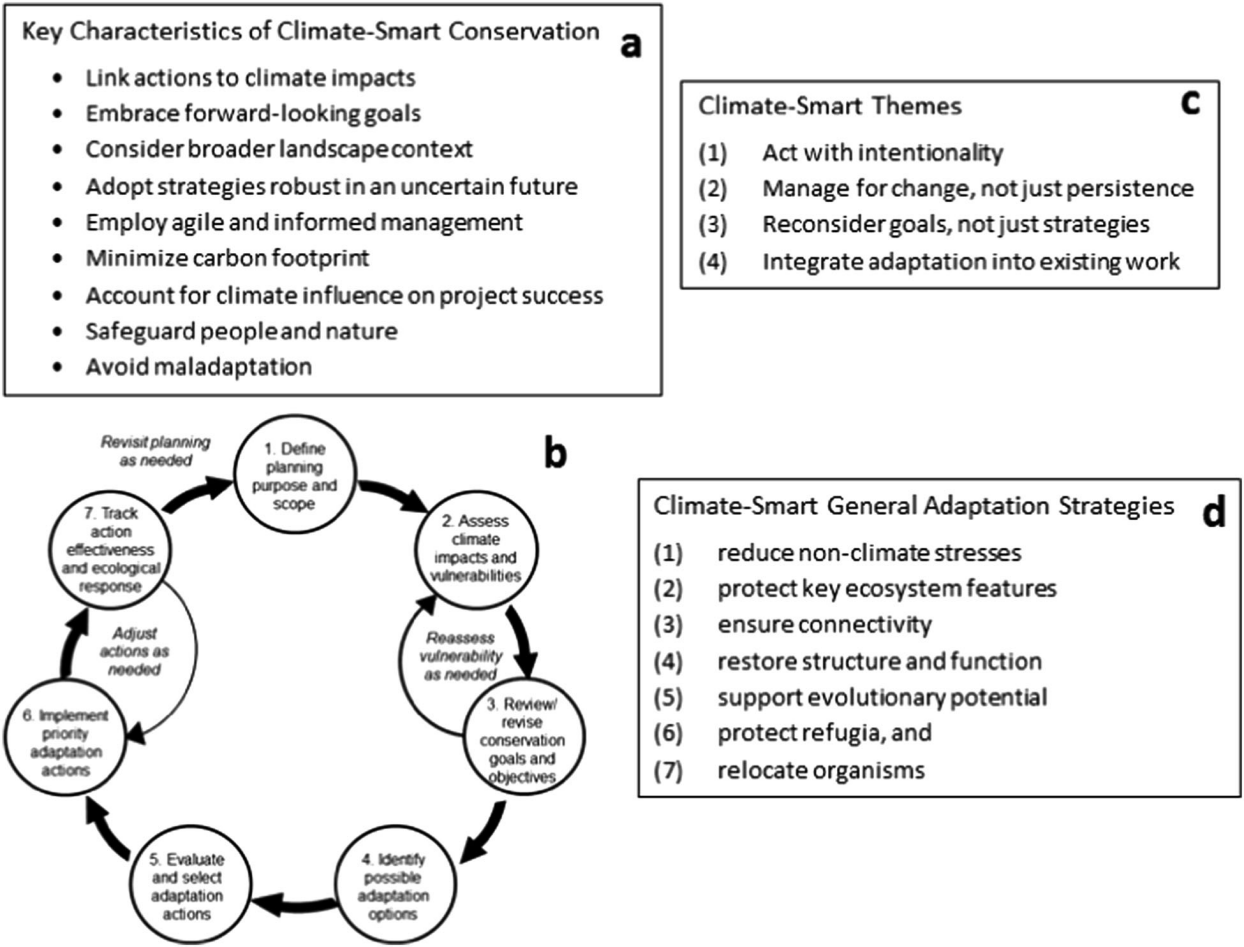
Climate-smart approach for adaptation planning and implementation. **a** Key characteristics of climate-smart conservation; **b** the climate-smart conservation (planning) cycle; **c** climate-smart themes; and **d** climate-smart general adaptation strategies (Stein et al. 2014)

The climate-smart approach includes: nine principles, or “key characteristics” (Fig. 1a) of climate-informed conservation; a generalized planning cycle (Fig. 1b) comprised of discrete steps that can be informed by climate-smart management; and four over-arching themes that characterize fundamental concepts of climate change adaptation (Fig. 1c). There is also a set of general adaptation strategies (Fig. 1d) presented as one framework for generating adaptation options in a way that embodies an ecosystem-based management approach (McLeod et al. 2005; United Nations Environment Programme (UNEP) 2011). This framework recognizes the importance of focusing management on sustaining ecosystem functions, processes, and services in order to protect ecological integrity and support ecosystem resilience. Maintenance of ecosystem resilience is a predominant paradigm for climate change adaptation, based on the premise that increasing resilience extends a system’s ability to cope with the added stress imposed by climate change (Bernhardt and Leslie 2013; Carilli et al. 2009; Fujita et al. 2013; Julius et al. 2008; McClanahan et al. 2012; Mumby et al. 2014; West et al. 2009; West and Salm 2003). Ecological resilience is defined as the ability of a system to absorb some degree of disturbance and persist within boundaries of a characteristic condition, to return to its original state after perturbation, or as the combination of “resistance” and “recovery” potential (Anthony et al. 2015; Cumming et al. 2005; Folke et al. 2002; Gunderson 2000; Holling 1973; Walker et al. 2004). While resilience is recognized as an important concept for adaptation to climate change threats, use of the term can at times be vague and used to justify any adaptation strategy in the continuation of a “business as usual” conservation approach. For effective climate change adaptation, resilience must be considered with rigor and explicitly linked to anticipated responses to climate change effects. At the same time, by taking the “long view” when considering climate change, climate-smart planning also recognizes the need to manage for ecosystem change in addition to persistence (Stein et al. 2014), because of the rate and magnitude of climate change and the potential for exceeding ecological thresholds (Stein et al. 2013).

While useful as a key starting point, the climate-smart conservation planning cycle and associated principles are general. For successful application to any particular natural resource, the generalized approach must be interpreted within the context of the particular ecosystem being managed, leading to an array of system-relevant adaptation options that address core objectives. We have targeted coral reefs as one of the first ecosystems for such “tailoring” and application of a climate-smart approach for several reasons. Coral reefs are complex “charismatic” ecosystems valued for their high biodiversity and productivity, their economic importance both commercially and recreationally, and the myriad other ecosystem services they provide (Cesar et al. 2003; Hoegh-Guldberg et al. 2007; Hughes et al. 2003; Moberg and Folke 1999). Coral reef scientists and managers have long been aware of ongoing coral reef degradation that has been occurring for decades if not centuries (Pandolfi et al. 2003), largely as a result of ecological disruptions caused by human influences (Jackson et al. 2001, 2014). Climate change is also having substantial interactive and cumulative impacts on reef health and stability (Buddemeier et al. 2004; Mumby and Steneck 2008). Changes in the climate drivers of such impacts are already measurable—ocean temperatures are warmer (by an average of +0.7 °C), pH is lower (−0.1 units), and carbonate ion concentrations are lower (~30 mmol kg^−1^) now than over the geologic record of 420,000 years (Hoegh-Guldberg et al. 2007). These changes have contributed to direct impacts such as increases in coral bleaching events (Bellwood et al. 2004; Brown 1997), reef dissolution along with reduced calcification and growth rates (Hoegh-Guldberg et al. 2007; Kuffner et al. 2013; Manzello et al. 2012; McLeod and Anthony 2012; Orr et al. 2005), and increased storm damage (Harmelin-Vivien 1994; Wilkinson and Souter 2008). Indirect effects include precipitation-driven changes in intensity and patterns of sediment and nutrient runoff that impair coral reef condition (Hughes et al. 2003; Richmond 1993), increased disease outbreaks (ICRI/UNEP-WCMC 2010; Work et al. 2012), and species range shifts that decouple ecological relationships (Greenstein 2006; Yamano et al. 2011).

The combination of high ecological and human-derived reef values, high levels of climate change impacts, and ongoing legacy of interactive human-induced threats make a compelling case for incorporation of effective climate-change adaptations into reef management plans. Threats to coral reefs due to climate change are substantial, and the future prognosis is poor if strong adaptation planning is not rapidly pursued (Buddemeier et al. 2004; Great Barrier Reef Marine Park Authority 2009; Hoegh-Guldberg et al. 2007). Adaptation is critically important to buy time for species and ecosystems (Hansen and Hoffman 2011), but should be seen as nested within a larger context of policies and actions to mitigate greenhouse gas emissions.

Despite the complexities, implementing climate change adaptations for coral reefs is not a futile endeavor. Coral reefs are not expected to inevitably disappear, but instead are likely to undergo substantial changes in species composition and community structure and function (Hughes et al. 2003). This expectation is based on a depth of adaptive capacity that is attributed to the diversity of corals and their symbionts (zooxanthellae), evidence for a range of responses to temperature and other stressors across these species, spatial and temporal variations in climate change, and the potential for human management (Berkelmans and van Oppen 2006; Buddemeier et al. 2004; Darling et al. 2013; Dixon et al. 2015; Guest et al. 2012). As a result, sufficiently extensive and well-conceived management of coral reefs aimed at increasing reef resilience could successfully preserve values and characteristics of coral reef ecosystems, even if there is uncertainty regarding the outlook for some ecosystem services over the long term (Hughes et al. 2003).

This paper presents initial results of the Corals and Climate Adaptation Planning (CCAP) project, which seeks to develop guidance for improving coral reef management through tailored application of the climate-smart approach. As a collaborative effort under the auspices of the Climate Change Working Group of the interagency U.S. Coral Reef Task Force, the CCAP project benefits from the expertise of a network of practitioners, managers and scientists from over a dozen Federal, State, and Territorial agencies, as well as local and national non-governmental organizations and academic institutions, to explore and test climate-smart adaptation planning principles specifically for coral reef management.

## Project Approach and Initial Focus

The CCAP project began with an evaluation of how well each step of the climate-smart cycle (Fig. 1b) is currently supported by existing tools, approaches, and best practices specific to coral reef management, achieved through a comprehensive, but not necessarily exhaustive examination of peer-reviewed journal articles, government reports, and grey literature. Pertinent literature was identified using relevant search terms in Google Scholar, Web of Science, and similar search engines. Representativeness of the literature obtained was assured by engaging a network of experts, who covered a broad range of technical expertise and geographic experience, to review our bibliography and provide additional sources, including case studies, management plans, and other unpublished information relevant to climate change adaptation in the context of coral reef management planning. Our experts network included climate scientists and coral reef and watershed scientists, managers, and practitioners knowledgeable of reef systems in the Caribbean, Hawaiian archipelago, Great Barrier Reef, and Pacific Island and Southeast Asian countries of the Coral Triangle region. In addition, the outputs of the project were guided and reviewed by the Project Technical Steering Committee under the Climate Change Working Group of the U.S. Coral Reef Task Force. Resources were mapped to one or more steps of the climate-smart cycle in order to (1) confirm the applicability of the generalized cycle to similar steps in coral reef management efforts, and (2) assess the “state of the science” and availability of tools for each step, again specifically for coral reefs.

**Fig. 2:**
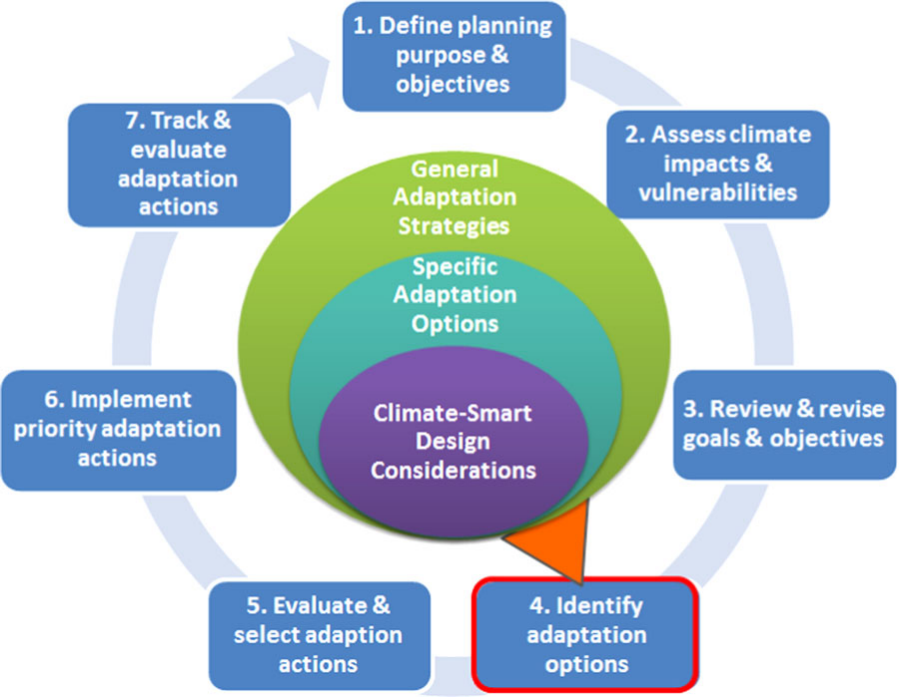
The climate-smart conservation cycle with the CCAP compendium framework

This led us to focus the first phase of the CCAP project on step four of the climate-smart cycle: identifying possible adaptation options (Step 4; Fig. 2). Earlier steps have an existing rich knowledge base of tools and methods (see, for instance, Dubois et al. (2011); Gitay et al. (2011); Glick et al. (2011); Strange et al. (2012); U.S. Environmental Protection Agency (EPA) (2012a, b)) that reflect the sophistication of the science and management community in setting clear management goals and assessing climate impacts and vulnerabilities with respect to those goals. Outputs from these approaches have informed discussion of a number of general adaptation strategies. However, moving beyond general strategies into specific adaptations for reef systems in particular places requires analysis of specialized actions under large uncertainties. There is limited guidance for managers on how to bridge this gap to develop specific, implementable actions that incorporate location-specific climate change concerns.

The CCAP project focused on filling this gap by building on one of the approaches for generating adaptation options presented in the Climate-Smart Conservation guide (West and Julius 2014), which in turn built on the U.S. Climate Change Science Program’s synthesis of adaptation options for climate-sensitive ecosystems and resources (U.S. Climate Change Science Program (CCSP) 2008). The result is the CCAP Compendium (see next section; full Compendium available as Supplementary Online Material), a framework of general strategies, coral reef-specific options, and climate-smart design considerations that managers can use as a resource from which to jumpstart their climate change adaptation efforts. A key advancement is the inclusion of “climate-smart design considerations” that help managers to move from fairly generic adaptation options to actionable alternatives for a particular place and situation. These concepts are presented in greater detail in the sections that follow.

## Building a Foundation: the CCAP Compendium

The CCAP Compendium (see Supplementary Online Material) is a resource for undertaking the identification of adaptation options for coral reef ecosystems. It includes seven general climate-smart adaptation strategies from the Climate-Smart Conservation guide (West and Julius 2014), which were developed to encourage a process of ecosystem-based “brainstorming” of options (Colls et al. 2009; Stein et al. 2014). Effective application is predicated on first demonstrating the relevancy and appropriateness of the general adaptation strategies for coral reefs, through the compilation of illustrative adaptation options for each strategy, and identification of climate-smart design considerations for each option.

### Confirming Relevancy of General Adaptation Strategies

The relevancy and appropriateness to coral reef ecosystems of the general adaptation strategies described below was determined by: (1) exploring their meaning and intent in the context of coral reef ecosystems (as presented in the following subsections), and (2) comparing them to strategies reported in the literature and promoted within existing management practices for coral reefs (through tabular comparison). This literature-based assessment was reviewed and supplemented with additional inputs from our participating coral expert group; additional stakeholder feedback on the relevancy of the strategies included in the Compendium was obtained through its trial application at a broader-based workshop (see section below on Applying the CCAP Compendium for details).

#### Reducing non-climate stressors

This strategy focuses on minimizing local-scale human-generated stressors that hinder the ability of species or ecosystems to withstand or adjust to climate events. Stressor reduction is intended to enhance ecosystem resilience (Kareiva et al. 2008) and is a management strategy commonly applied to coral reefs (Burke et al. 2011; The Nature Conservancy 2015). More than 60 percent of the world’s reefs are under immediate and direct threat from local activities such as overfishing and destructive fishing, coastal development, watershed-based pollution, or marine-based pollution and damage (Burke et al. 2012; Fernandes et al. 2012; Jackson et al. 2014). Climate change exacerbates these local stressors and increases sensitivity to other stressors (McLeod et al. 2012; West and Julius 2014). Ocean acidification and coral bleaching from ocean warming reduce reef calcification and increase sensitivity to disease and other local threats (McLeod et al. 2012). Watershed-based nutrient pollution can also make reef species more susceptible to climate impacts by reducing bleaching thresholds (D’Angelo and Wiedenmann 2014) and increasing disease severity (Bruno et al. 2003). Because actions under this strategy are already widely used, extra emphasis needs to be placed on considering climate-smart design within existing management portfolios.

#### Protecting key ecosystem features

This concept addresses management of the structural characteristics, organisms, and areas that play a critical role in maintaining resilience in the current or future ecosystem of interest (West and Julius 2014). For instance, displacing or removing a population of keystone species usually results in the re-organization of the ecosystem and sometimes results in its collapse (Jackson et al. 2001; Keller et al. 2009). Marine protected areas are widely used for protecting key ecosystem features (Keller et al. 2009). Key functional groups such as herbivores are protected through catch and size restrictions to support reefs that are threatened by algal domination (Hawai’i Administrative Rules (HAR)) or recovering from disturbances such as hurricanes and coral bleaching events (Edwards et al. 2010). However this type of option is less frequently implemented than some research recommends (Bohnsack et al. 2000). While many coral reef bleaching response plans highlight the need for protection of herbivores, few managers have or use statutory authority to impose emergency rules that could place temporary restrictions on herbivore fishing. The impacts of climate change on life history and recruitment of keystone species and functional groups need to be evaluated in order to protect those features that can confer resilience to future changes.

#### Ensuring connectivity

Ensuring connectivity to facilitate movement of energy, nutrients, and organisms is a key aspect of maintaining ecosystem function that is highly relevant in the climate change context (Lindenmayer et al. 2008). As an adaptation strategy, incorporating physical connectivity supports genetic exchange among subpopulations of marine organisms, particularly at the spatial and temporal scales over which marine populations are connected by larval dispersal (Cowen et al. 2007; Cowen and Sponaugle 2009). The linking of local populations through the dispersal of individuals as larvae, juveniles or adults, is a key factor to consider in marine reserve design, since it has important implications for the persistence of meta-populations and their recovery from disturbance (Green et al. 2014). The impacts of climate change on both the reefs that serve as sources of recruits and the ocean circulation that delivers the larvae needs to be considered in designing networks of marine protected areas (Fernandes et al. 2012).

#### Restoring ecosystem structure and function

Here the emphasis is on rebuilding, modifying, or transforming ecosystems that have been lost or compromised, in order to restore desired structures and functions (West and Julius 2014). Restoration can focus on restoring intact ecosystems or characteristic species complexes that are important to the resilience of the system (Kareiva et al. 2008). This is consistent with the common goal of supporting continuation of diverse and functioning ecosystems for sustainable use (Clean Water Act 1972; Glick et al. 2011). The existing species composition may not persist under a changing climate, but there is the potential to preserve key ecosystem services. Examples of management practices to restore ecosystem structure and function include restoring herbivorous fish and invertebrate populations, preventing and managing invasive species, controlling outbreaks of coral predators, and re-establishing source populations of corals. For instance, herbivores functionally contribute to reef recovery following disturbances such as hurricanes and coral bleaching events (Edwards et al. 2010). Conversely, invasive species proliferate from changes in ocean currents and increasing stress on reefs. This can transform marine habitats by displacing native species, changing community structure, and altering fundamental processes such as nutrient cycling and sedimentation (Molnar et al. 2008). Note that there is overlap among activities in this and several of the other strategies, especially protecting key ecosystem features.

#### Protecting refugia

This strategy involves identification and protection of areas less affected by or more resilient to climate change as sources of “seed” for recovery or as destinations for climate-sensitive migrants (West and Julius 2014). In coral reef ecosystems, refugia may be identified by coral species resistant to sea surface temperature anomalies and areas that support oceanographic and biogeochemical conditions that ameliorate the impacts of increased sea surface temperature, ocean acidification, and other impacts of climate change (Manzello et al. 2012; McClanahan et al. 2011; Storlazzi et al. 2013; van Hooidonk et al. 2013). Refugia may also be found in areas where the distribution of coral reefs is expanding or projected to expand poleward due to increasing sea surface temperatures (Baird et al. 2012). Such refugia need to be identified and incorporated into marine protected area design (Fernandes et al. 2012; Keller et al. 2009).

#### Relocating organisms

Relocating organisms refers to human-assisted transplantation or translocation of corals or other organisms from nurseries or other reefs to overcome environmental barriers and negative chemical cues that can impede recruitment. Establishing nurseries and transplanting corals with thermotolerant symbionts from the southern Persian/Arabian Gulf to reefs in the Indian Ocean could facilitate adaptation to the higher water temperatures expected in the future (D’Angelo et al. 2015). Negative chemical cues from degraded reefs with seaweeds may serve as barriers to recruitment, requiring transplantation of corals to provide positive chemical cues that attract recruits (Dixson et al. 2014). Climate-induced changes in currents and associated connectivity (refer to connectivity section for more detail) can also disrupt recruitment patterns and might necessitate “restocking” organisms to critical reefs that are now cut off. Transplantation as an adaptation strategy would also incorporate the emerging discussion and research on human-assisted evolution (see section below on Supporting Evolutionary Potential). Although early translocation attempts sometimes fell short of achieving desired objectives (Bentivoglio 2003), this strategy is now receiving more attention.

#### Supporting evolutionary potential

This concept centers on protecting a variety of species, populations, and systems in multiple places to hedge against losses from climate disturbances, and managing these systems to assist positive evolutionary change (West and Julius 2014). The concept of “risk spreading” is central to climate change adaptation. It can be captured through representation by different forms of species, ecosystems, or habitats (Kareiva et al. 2008), essentially preserving existing diversity at multiple levels (genetic, organismic, etc.). It is also captured through replication—preservation of multiple examples of habitats, populations, or ecosystems (Kareiva et al. 2008), in this case to address climate change risks and support positive biological adaptation. In coral reef ecosystems, representation and replication of habitat types through networks of marine protected areas spreads risk in the face of uncertainties (Fernandes et al. 2012; Kareiva et al. 2008) and maintains genetic diversity that provides the raw material for evolutionary change (Kareiva et al. 2008; West and Julius 2014). Populations in different locations may contain distinct genetic mixes that represent adaptation to different sets of local conditions. Genetic diversity in corals and their symbionts may reduce bleaching and prevent reef collapse (Barshis et al. 2013; Baskett et al. 2009), and some research suggests evolutionary adaptation is already occurring (Logan et al. 2014). Proposed assisted evolution approaches such as inducing acclimatization, modifying microbial symbiont communities, and selective breeding (van Oppen et al. 2015) are very new approaches to identify and propagate species with climate-resistant genetic variants and intra-generational acclimatization in much the same way as plants are bred for resistance or tolerance to climate and pests. Sometimes using these techniques may require human-assisted relocation/transplantation (see section above on Relocating Organisms). Given the very recent and evolving experience with such “assisted” techniques, additional research will provide insights into their feasibility and potential impacts.

The seven general adaptation strategies described are quite applicable to coral reef ecosystems, as demonstrated by the coral reef management literature. In addition, the strategies were largely consistent with other coral reef-specific frameworks (Table 1. For example, Fernandes et al. (2012) identified five overarching strategies for designing resilient networks of marine protected areas that integrate fisheries, biodiversity, and climate change objectives for coral reefs. Meanwhile, management strategies promoted for conservation practitioners in the Reef Resilience Coral Reef Module (The Nature Conservancy 2015) emphasize managing local stressors and establishing marine protected areas and networks. And while the categories used by The Nature Conservancy are largely organized by type of action or by target stressor, the integration of ecosystem-focused principles is preserved in the elaboration of these strategies (The Nature Conservancy 2015). All three frameworks have some key strategies in common (e.g., reduction or management of non-climate stressors/threats), and the full range of option types is captured using the climate-smart strategies.

**Table 1:** Comparison of general adaptation strategies

General adaptation strategies for multiple ecosystem types (Stein et al. 2014; West and Julius 2014)	Coral reef specific strategies for multiple conservation objectives (Fernandes et al. 2012)	Coral reef specific strategies for multiple conservation objectives (The Nature Conservancy 2015)
A. Reduce non-climate stresses	• Threat reduction	• Manage local stressors
		• Reduce land-based impacts
		• Manage fisheries
B. Protect key ecosystem features	• Protect critical areas	• Establish marine protected areas
C. Ensure connectivity	• Incorporate connectivity	• Establish marine protected areas networks
D. Restore structure and function	• Sustainable use	• Facilitate passive restoration
		• Manage for social resilience
E. Protect refugia	• Protect critical areas	• Manage for ocean acidification
F. Relocate organisms	N/A	• Conduct active restoration
G. Support evolutionary potential	• Risk spreading	• Establish marine protected areas networks

Based on this review and stakeholder feedback, the Compendium (see Supplementary Online Material) maintains the seven general adaptation strategies as a relevant and useful framework for identifying adaptation options for coral reef ecosystems. It is not expected that all seven strategies will be explicitly included in each and every site-specific plan. Rather, they are intended to serve as a framework for brainstorming potential new adaptation options to fill gaps, or to refine management goals and objectives and refocus management efforts with a forward-looking, climate change perspective. To the extent that some potential climate change adaptation strategies may not be perceived as fitting well into one of these seven categories, such as shading of corals for temperature mitigation (Rau et al. 2012), it must be emphasized that the value of the Compendium and its structure of seven ecologically oriented strategies is in its potential to stimulate the brainstorming process, and should not constrain the possible inclusion of novel approaches. In reflecting an ecosystem-based approach to adaptation, it also incorporates traditional conservation strategies, such as reducing non-climate stressors. However, it becomes clear that especially the more forward-thinking ecological strategies (e.g., restoring ecosystem structure and function, supporting evolutionary potential, protecting refugia, and relocation) should be further refined with an explicit climate-change focus. Finally, the strategies are not mutually exclusive and may be used in combination.

### Compiling Adaptation Options for Coral Reefs

Building on the general adaptation strategies as a framework for the Compendium, over 250 peer-reviewed articles and other sources of information such as guides and case studies were reviewed and mined for illustrative, coral-specific adaptation options. These adaptation options were then binned into the seven general adaptation strategies that make up the Compendium. An excerpt of the Compendium is provided in Table 2, showing one example option for each of three strategies. The full list of adaptation options for each of the seven strategies in the Compendium can be found in the Supplementary Online Material.

**Table 2:** Excerpt from the CCAP compendium showing selected general adaptation strategies, adaptation options, and design considerations (see Supplementary Online Material for full list)

General adaptation strategies and adaptation options	Climate-smart design considerations
*A. Reduce Non-Climate Stresses*	
Minimize localized human stressors (e.g., pollution, fishing pressure) that hinder the ability of species or ecosystems to withstand or adjust to climatic events	
i. Minimize land-based pollution due to excessive loadings of suspended sediments and nutrients from agriculture, deforestation, urbanization, and other land uses	• How will climate change-related shifts in precipitation patterns and hydrology affect runoff of sediments and nutrients from different land use types to coastal waters?
	*•* How and in what locations could the protection or restoration of forests and/or wetlands, the management of agricultural areas and/or roads, or the installation of land-based pollution controls be focused to minimize runoff to coastal waters?
	*•* How will any such pollution control installations have to be designed (including size, structural characteristics) and located to both accommodate projected sediment or nutrient runoff loads and also withstand the direct physical climate change impacts of larger, more intense storms, greater erosion, etc.?
*B. Protect Key Ecosystem Features*	
Focus management on structural characteristics (e.g., geophysical stage), organisms, or areas (e.g., spawning sites) that represent important “underpinnings” or “keystones” of the current or future system of interest	
i. Manage functional species and groups necessary for maintaining the health of reefs and other ecosystems	• What is the vulnerability of functional species and groups (e.g., herbivores, apex predators) to the interaction of climate change with other human and natural stressors, and in what locations are they most vulnerable?
	• What management options can be employed, and in which locations, to minimize impacts on the most vulnerable species and groups?
*C. Ensure Connectivity*	
Protect and restore habitats that facilitate movement of organisms (and gene flow) among resource patches	
i. Identify and manage networks of resilient reefs connected by currents	• Which areas have demonstrated resistance to/or recovery from exposure to climate change impacts?
	• Which areas are projected to have less exposure to climate change impacts (e.g., increased sea surface temperatures, decreased ocean pH) and could therefore serve as refugia?
	• How will climate change affect currents that provide connectivity between resilient areas?
	• What are the implications of this information for design of managed area networks to maximize connectivity and maintain it into the future?

The process of compiling and binning adaptation options represents Step 4 of the Climate-Smart Cycle and is intended to support “brainstorming” on the part of managers as they review (or develop new) management plans and revise them to be climate-smart. Our literature review resulted in three to eight adaptation options identified for each strategy. Some options could be categorized under more than one strategy, i.e., the strategies are not mutually exclusive. This provides a beneficial degree of overlap and redundancy, to ensure that all options are captured from a variety of strategy angles.

In the first example (Table 2), the option under *Reduce Non-Climate Stresses,* “minimize land-based pollution”, came up in many coral reef management references. Options dealing with other anthropogenic stressors such as fishing pressure, shoreline hardening structures, direct habitat destruction, and non-land based pollutant discharges were also identified. The range of familiar options included under this strategy (see the full Compendium in the Supplementary Online Material) reflects the long history of coral reef management activities that have focused on pollution reduction, even prior to the addition of climate change as a management concern.

In the second example (Table 2), options such as this one under *Protect Key Ecosystem Features,* “manage functional species and groups”, are diverse. This one addresses protection of functional groups that are key within the coral reef ecosystem. But this strategy also includes options that address components outside of—but functionally linked to—the coral reef system (e.g., wetlands/mangroves, seagrass beds, etc.). It encompasses protection or management of unique areas, sites specific to life cycle functions, critical habitat for threatened or endangered species, or areas of high diversity.

The third example, under *Ensure Connectivity*, “identify and manage networks of resilient reefs”, recognizes the ecological connections among reef patches that can be important to the flow of organisms for recruitment and gene flow. The example option represents one possible component of protecting such connectivity into the future by identifying networks of resilient reefs. Other options under this strategy include identifying ecological connections among areas, or protecting up-current reefs as potential sources of organisms and propagules. Protection of different types of habitat diversity (e.g., including protection of multiple habitat types, reef areas of different sizes and shapes, etc.) is another theme incorporated in the options aimed at preserving ecological connectivity. Options in this strategy have some overlap with other strategies. For example, the protection of habitat areas critical as source populations can also represent the preservation of key ecosystem features, or support of evolutionary potential.

### Developing Climate-Smart Design Considerations

The general adaptation strategies and example adaptation options in the Compendium provide ideas for identifying adaptation options; however, for any particular adaptation option to be considered climate-smart for the management of a specific reef, it needs to explicitly address vulnerabilities of the conservation targets in that specific place. To achieve this, each adaptation option needs to be subjected to “climate-smart design considerations” (see examples in Table 2). Addressing the climate-smart design considerations is the process through which management actions are configured to account for climate change effects, key vulnerabilities, and their interactions with the other stressors. Answering the climate-smart design questions for a candidate adaptation option is aimed at developing enough information to determine how, when, and where a management action should be adjusted to be responsive to and effective under the combination of site-specific climate change impacts and stressor concerns. Climate-smart design considerations fall into two general categories:

- How will climate change directly or indirectly affect how stressors impact the system, including through effects on stressor interactions*?*
- What are the implications of this information for the location, timing, or engineering design of management actions?

The Compendium provides examples of climate-smart design considerations in both categories, to focus refinement of adaptation options to account for future as well as current conditions and make explicit links to climate-related impacts and vulnerabilities (Table 2; see Supplementary Online Material for full list). A management action designed to reduce land-based pollution from agriculture needs to account for both historical conditions in precipitation and hydrology and future conditions that will result from climate change. Projected changes in the distribution and intensity of rainfall may require updated design specifications to enable more stringent pollution control and forest and wetland management practices. An understanding of the impacts of climate change on the life history and vulnerability of herbivore species is needed to design management actions to protect this key ecosystem feature now and in the future. Climate change may result in changes in ocean currents that could affect recruitment and connectivity. The design of marine protected area networks needs to incorporate consideration of future oceanographic conditions to facilitate gene flow and habitat connectivity.

The example adaptation options and climate-smart design considerations in the Compendium are meant to be illustrative rather than comprehensive and to stimulate thinking about site-relevant possibilities. As new research and practices emerge, the range of examples will continue to grow, and the Compendium will need to be reviewed and updated over time.

## Applying the CCAP Compendium

Integral to the development of the CCAP Compendium and the process of using it is recognition that many coral reef areas have conservation plans already in place or under development, reflecting varying degrees of thought about possible climate change impacts. Accordingly, the Compendium is designed so it can be referenced during the revision of existing plans, but is equally applicable in a *de novo *planning process.

**Fig. 3:**
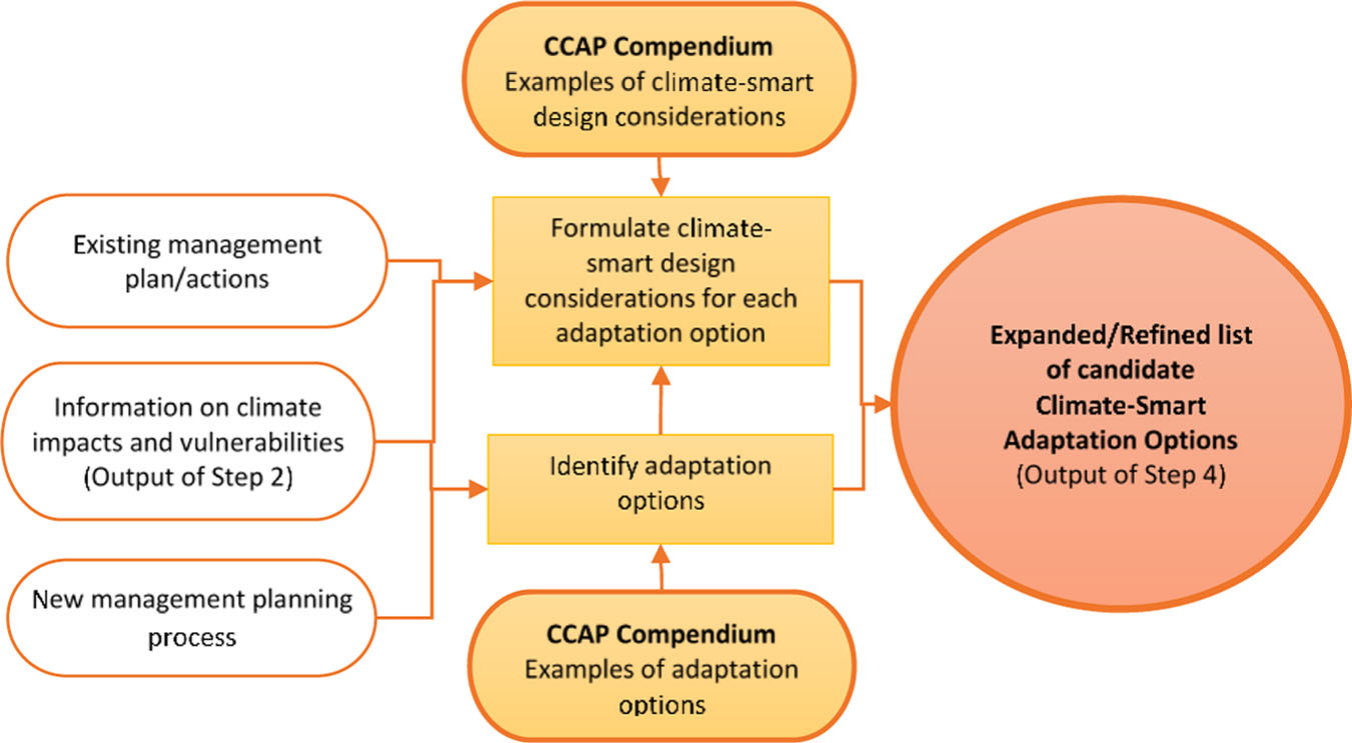
Flow chart for using the CCAP compendium in step 4 of the climate-smart conservation cycle

Figure 3 shows how the Compendium may be used to revise or expand a set of existing management actions. For revision of a plan, the list of existing actions can be compared to the Compendium and categorized according to the strategies they address. Then the Compendium can be reviewed to identify potential gaps, guide brainstorming to fill those gaps, and/or identify strategies that may no longer make sense in the context of anticipated climate change effects. Any additional candidate adaptation options would be added to the existing list. Then, climate-smart design consideration questions are formulated for each option on the expanded list. At this point, the outcome of the brainstorming component of Step 4 is intended to be a broad set of potential adaptation actions that are responsive to the range of climate change impacts and vulnerabilities that have been identified for a particular site (West and Julius 2014). These are associated with a set of climate-smart design questions that, once answered, will more explicitly link the design of that option to the relevant combination of climate change and other stressor impacts that the option is intended to address.

A stakeholder workshop was held to test this process and assess its efficacy in assisting practitioners to brainstorm and refine specific, place-based adaptation actions and craft associated climate-smart design considerations. The workshop was held in Honolulu, Hawai’i, and focused on West Maui’s coral reefs as a test case using the Wahikuli-Honokōwai Watershed Management Plan (Sustainable Resources Group Intnl 2012) and Conservation Action Plan (Hawaii Department of Land and Natural Resources et al. 2014). Input and feedback from participants was obtained through facilitated discussion during structured exercises undertaken with participants in two break-out groups, and was captured as a synthesis of discussions, as related and opposing inputs, and as a tabulated series of category-specific inputs. To support this test effort, participants were given a desk-top vulnerability assessment using existing climate change information for the case study area (Box 1). Participants included 22 experts in coral reef management and science, especially from West Maui, the broader Pacific region, and the Caribbean, but including representation from major managed coral reef systems globally (e.g., the Great Barrier Reef, American Samoa, Palau, Guam, Northern Mariana Islands); and with representation from Federal, State, and Territorial agencies as well as local and national non-governmental organizations and academia.

**Table 3:** Examples from the stakeholder workshop, based on a case study using West Maui management plans

Action			Climate-smart design consideration
1	Existing	Install water bars, terraces, and microbasins in dirt roads in agricultural areas	How will increasingly severe storms affect the volume of runoff onto the near shore coral reef? How can the design be adjusted to account for these effects?
	Modified	Install terraces adjacent to dirt roads in agricultural areas to reduce sediment/nutrient loads by x and y percent	How will increasingly severe storm events, in combination with increasingly frequent dry periods, affect the volume of runoff onto the near shore coral reef? What will be the spatial pattern of these effects with respect to the location of dirt roads in agricultural areas? How will the design of terraces need to be adjusted to: place them at locations of worst erosion; ensure their capacity to effectively reduce sediment/nutrient loads by x and y percent; and account for how maintenance and replacement schedules would need to change?
2	New	Protect and manage adjacent (Olowalu) coral reef areas that are connected hydrodynamically and can serve as recruitment sources for coral reefs in West Maui	How will climate change affect connectivity of downstream reefs to Olowalu areas that are recruitment sources? How will climate change affect stressors to be managed in Olowalu areas (pollution, bleaching, disease, reduced calcification)? What are the implications of this for how we prioritize, replicate, represent and increase level of protection of Olowalu areas, possibly at a greater scale?

Original Action #1 and design considerations developed in advance of the workshop are compared with modifications that reflect the results of the workshop exercise. “New” action #2 was identified as a gap by participants after reviewing the Compendium

Table 3 provides an excerpt from a larger table of case study actions and design considerations reviewed by stakeholders at the Honolulu workshop. Action 1 is an example of an existing action drawn directly from the West Maui management plan. Initial draft climate-smart design considerations were developed for presentation to stakeholders during the workshop. Through discussions with the participants in the workshop, “modified” versions of actions and climate-smart design considerations emerged. Action 2 was developed by the participants as a “new” action inspired by the Compendium to fill a perceived gap in addressing climate vulnerabilities. This new management action combines elements of several strategies and options in the Compendium (e.g. B. i., C. iv., C. vi.; see Supplementary Online Material) and refines them into a place-based action specific to the West Maui context.

**Box 1:** Vulnerability Exercise for Stakeholder Workshop

Since information on climate impacts and vulnerabilities is one of the inputs needed in Step 4 of the climate-smart cycle, a desktop vulnerability assessment was developed as a resource for the participants. While there are many methodologies that could be chosen to complete a vulnerability assessment for a site, for illustrative purposes we used the LEAP guide: “Climate Change Adaptation for Coral Triangle Communities: A Guide for Vulnerability Assessment and Local Early Action Planning” (U.S. Coral Triangle Initiative Support Program, 2013). The LEAP method was applied to develop a vulnerability assessment based on climate information and projections for the main Hawaiian archipelago and information on threats to reefs in the watershed management and conservation action plans for West Maui. This desktop assessment was provided and used hand in hand with the CCAP Compendium at the workshop.	

Participant feedback indicated that using the Compendium encouraged practitioners to clarify options, making them more specific in terms of intended action and more clearly related to their target stressors. Action #1, the installation of water bars, terraces, and microbasins, was considered important but was found to be too general for discussion because the action included several components (water bars, terraces, etc.) that would have climate-smart design considerations. As a result, the action was refined to focus only on terraces, and the other techniques would each be assessed separately as additional actions. The iterative process of considering the action, and then its design considerations, led stakeholders to modify and refine the design considerations, including incorporation of more specific climate change impacts that would affect the target stressor and various temporal considerations such as how the life cycle of the action compares to the timing of climate change effects.

In the case of Action #2, the workshop participants could see the value of adding this new option, which was focused on preserving connectivity. Through review of the Compendium examples and comparison to issues characterized for the West Maui reefs and to actions already included in their existing management plans, they concurred that this option addressed gaps in their plan. This new option resulted in expanding the geographic scope of the management area to an adjacent reef (*Olowalu*) deemed important as a source of coral recruitment to downstream reefs of West Maui. This also led to considerable refinement of the design considerations to reflect this specificity of reef type and place.

Overall, participants in the Honolulu workshop thought applying the Climate-Smart Cycle to coral reefs was valuable. There was also an emerging appreciation for how developing outputs in Step 4 often led to recognizing needs for additional, more detailed, or more clear information from previous steps, reinforcing the iterative nature of the process. The Compendium was considered a rich resource for adaptation ideas. There was particular interest in the concept of climate-smart design considerations, which promotes the idea that rigorous adaptation must be specifically linked to the when, where, and design of an option. Only by considering climate-smart design can managers develop options that address specific place-based information on the combination of climate change with other stressor impacts. By delving into the West Maui example, participants found that it wasn’t necessarily easy to develop meaningful design considerations, but it was essential.

## Discussion and Conclusions

Building on the climate-smart conservation cycle and general adaptation strategies of Stein et al. (2014), a new tool, the CCAP Compendium, was developed to advance the ability of coral reef managers to integrate climate change thinking into management planning and facilitate effective implementation of climate change adaptations for coral reefs. The Compendium provides a framework and guiding examples of coral-reef specific adaptation options to help reef managers refine existing or develop new adaptation options within the context of their ongoing management planning processes. The formulation of climate-smart design considerations for each option establishes a thought process that explicitly links climate change impacts to coral reef management. This creates a bridge for managers to move from a “business as usual” or “more is better” design of management actions, to revision of actions so their designs accommodate a range of plausible future conditions of the reef driven by climate change.

As a new instrument for implementing Step 4 of the Climate-Smart Cycle (Fig. 2), a step which has heretofore received little operational attention, use of the Compendium, including development and application of climate-smart design considerations, requires inputs from earlier climate-smart planning steps. Decision makers engaged in defining the planning purpose and objectives (Step 1) and assessing climate change impacts and vulnerabilities (Step 2) have numerous tools and processes to help them accomplish these steps (e.g., Dubois et al. (2011); Gitay et al. (2011); Glick et al. (2011); Strange et al. (2012); U.S. Environmental Protection Agency (EPA) (2012a, b)). The Compendium tool and process presented here integrates with and utilizes outputs from these existing tools, extending their value to managers and decision-makers.

Limitations of this tool for coral reefs include the current lack of research and development of techniques for less widely applied strategies, such as Supporting Evolutionary Potential or Relocating Organisms. As previously noted, this highlights the need for review and expansion of the Compendium as additional research and new information become available. Similarly, as practical applications of the Compendium and framework expand, the management experience gained will provide valuable insights into the effectiveness of—and improvements needed in—adaptation design. The application of this framework also assumes a relatively structured planning and decision-making process that includes the development of site- and resource-specific climate change vulnerability information. We recognize that commonly occurring limitations in resources, such as time and funding for management planning, can constrain the level at which inputs needed for the application of this framework can be developed. That said, the Compendium provides a clear starting point, thought process, and scientific basis for proceeding with climate-smart design using the best currently-available information, while also recognizing the need for future expansion and improvement as new knowledge becomes available.

**Fig. 4:**
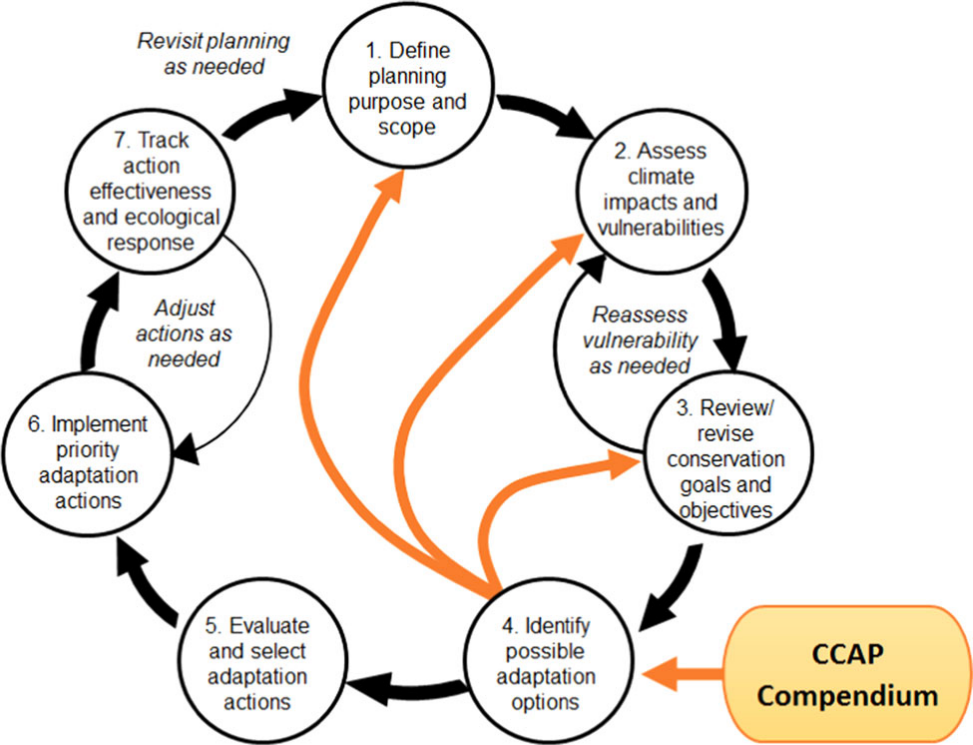
Additional feedback loops in the climate-smart conservation cycle

Using the Compendium to identify adaptation options also provides valuable insights that make it advantageous to revisit earlier steps in the climate-smart cycle before advancing to evaluation and selection in Step 5 (Fig. 4). For example, a manager may need to modify the geographic scope and scale of the plan (Step 1) if the expanded list of adaptation options incorporates connectivity with other sites outside of the managed area. Or, the expanded list of candidate climate-smart adaptation options may cause managers to revisit conservation goals and objectives (Step 3), for example if the management focus shifts from protection of key ecosystem features to managing for ecosystem services. Finally, the formulation of climate-smart design questions may reveal gaps in knowledge that lead to additional vulnerability assessment (Step 2). In some cases the necessary site-specific vulnerability information may exist; in others managers may need to decide whether gathering such information is important enough to their decision to be worth the requisite time and money. In making these decisions, it is worth considering how additional information could help in understanding the relative risks and benefits of protecting reefs with the highest vulnerability vs. those with low or medium vulnerability, where human intervention may make the biggest difference.

Uncertainty and variability in projections of future climate conditions are realities that must be embraced in our planning framework in order to understand and manage risks as part of designing and selecting adaptation strategies (Dessai et al. 2009; Hoffman et al. 2014; Hulme et al. 2009; Johnson and Weaver 2009). To the extent possible, it is desirable to formulate actions that are robust to addressing uncertainty, e.g., that can be successful under a wide variety of climate changes (Brown 2011; Kareiva et al. 2008). Being “climate-smart” involves asking the key questions (design considerations) about the impacts of climate change that are of particular concern relative to existing conditions of the target reef and the management options being considered. By asking these questions, a clearer picture forms of gaps in the information needed to characterize climate change threats particular to the reef being managed. Once this information is obtained, insights emerge as to whether the options under consideration can accommodate that level of threat and reduce risks, and what modifications (e.g., in design, placement, timing, etc.) will be needed to do so effectively. In particular, looping back to Step 2 (Fig. 4) in order to develop more specific types and scales of climate vulnerability information provides the opportunity for the iterative process of making coral reef management climate-smart. It is recognized that much of the uncertainty in climate projections is irreducible (Pruyt 2007). Thus it is the range of climate projections relevant to the management options being evaluated that are used to judge and improve the robustness of the adaptations being considered, while at the same time clearly defining assumptions and risks when using the best available data. Development of robust, climate-smart adaptation options may come at a cost, and may even be prohibitive or infeasible, but the information gathered in this iterative CCAP framework becomes a foundation for addressing such evaluation and selection criteria in subsequent planning steps (Fig. 4, Step 5). Further, the information can immediately begin to inform any periodic adaptive management process or planning cycle (e.g., watershed planning) where climate change may not have been a focus in the past, but is now identified as a priority issue to consider in planning.

The overall goal of Step 4 is to be able to develop and carry forward an expanded list of candidate climate-smart adaptation actions for evaluation and priority selection (Step 5, Fig. 4) and ultimately implementation (Step 6). The climate-smart design considerations formulated for each candidate adaptation option contribute to this goal directly by guiding the revision of options so they more effectively reduce climate change impacts and better withstand the direct impacts of climate changes that are anticipated for a site. In doing so, addressing the climate-smart design questions also provides information relevant to many common evaluation criteria (for example, effectiveness, feasibility, ability to fulfill management objectives). Thus evaluation and selection of actions should be done only after climate-smart design questions have been addressed. Stakeholders at the Honolulu workshop recognized the value of the climate-smart design considerations as a mechanism for linking site-specific climate vulnerabilities to the design of adaptation options for the targeted site considering the suite of human stressors and uses on the site. They also recognized that the process is complex and articulated the need for a more explicit, step-by-step guided process for answering, or “unpacking” the questions once formulated. Ongoing efforts of the CCAP project are to develop this process and an associated tool to aid in the unpacking of climate-smart design considerations.

The Climate-Smart Conservation guide (Stein et al. 2014) sets forth key characteristics and themes, a planning cycle, and general adaptation strategies that could be applied to both terrestrial and aquatic ecosystems. Step 4 of the cycle served as a useful entry point to apply the use of this framework to a specific ecosystem—coral reefs. The general adaptation strategies (West and Julius 2014) serve as a robust framework for coral reef systems to identify gaps in existing management plans for climate-smart adaptation. The process of developing climate-smart design considerations highlights uncertainties that might lead to revisiting steps in the climate-smart cycle and refining actions to better manage risks under future conditions. Overall, the insights gained through this coral reef-specific application of the climate-smart conservation framework illustrate its applicability and relevance to resource management in general.

## Electronic supplementary material

Supplementary Material
