# Supplementary material for: Climate-Smart Design for Ecosystem Management: A Test Application for Coral Reefs
Source: Environ Manage. 2016 Oct 12;59(1):102–17. doi: 10.1007/s00267-016-0774-3 (PMC5219003; doi:10.1007/s00267-016-0774-3)
Supplement: Supplementary file 1 — Supplementary Material [file 267_2016_774_MOESM1_ESM.docx]

**Supplementary Material**

**Journal: Environmental Management**

**Article: Climate-smart design for ecosystem management: A test application for coral reefs**

Authors:

Jordan M. West (Corresponding author)

Office of Research and Development, U.S. Environmental Protection Agency, 1200 Pennsylvania Ave, NW (8601P), Washington, DC 20460 U.S.A. Email: west. jordan@epa.gov; Phone: 703-347-8584; Fax: 703-347-8694

Catherine A. Courtney, Tetra Tech, Inc., 737 Bishop St., Suite 2340, Honolulu, HI 96813-3201 U.S.A.

Anna T. Hamilton, Tetra Tech, Inc., Center for Ecological Sciences, 502 W. Cordova Road, Suite C, Santa Fe, NM 87505 U.S.A.

Britt A. Parker, The Baldwin Group, Inc., NOAA Coral Reef Conservation Program, SSMC4, N/OCM6, Rm 10329, 1305 East West Hwy, Silver Spring, MD 20910 U.S.A.

Susan H. Julius, Office of Research and Development, U.S. Environmental Protection Agency, 1200 Pennsylvania Ave, NW (8601P), Washington, DC 20460 U.S.A.

Jennie Hoffman, Private Consultant, 4755 Northeast Lambs Lane, Poulsbo, WA 98370 U.S.A.

Karen H. Koltes, Office of Insular Affairs, MS 2429, U.S. Department of the Interior, 1849 C St. NW, Washington, DC 20240 U.S.A.

Petra MacGowan, The Nature Conservancy, 74 Wall Street, Seattle, WA 98121 U.S.A.

| CCAP Compendium: General Adaptation Strategies, Adaptation Options, and Climate-Smart Design Considerations^^[[1]](#footnote-2)^^ | |
| --- | --- |
| **General Adaptation Strategies[1] and Adaptation Options** | **Climate-Smart Design Considerations** |
| 1. **Reduce Non-Climate Stresses**   *Minimize localized human stressors (e.g., pollution, fishing pressure) that hinder the ability of species or ecosystems to withstand or adjust to climatic events* | |
| 1. Restrict future coastal development and shoreline modification, including hard coastal engineering and land reclamation [2, 3] | - *How will sea level rise and changes in the intensity and frequency of large storms affect coastal hydrology and erosion?* - *Given this information, how can the design, location and timing of development be planned or modified in order to minimize negative impacts to adjacent reefs?* |
| 1. Remove existing structures that harden the shorelines to allow inland migration of sand and vegetation [2, 3] | - *How will sea level rise and changes in the intensity and frequency of large storms affect coastal hydrology and erosion?* - *Given the above information, which structures should be the highest priority for removal in order to allow more natural migration of sand and vegetation?* |
| 1. Minimize land-based pollution due to excessive loadings of suspended sediments and nutrients from agriculture, deforestation, urbanization, and other land uses [2, 4-11] | - *How will climate change-related shifts in precipitation patterns (e.g. storm frequency and intensity) and in hydrology affect the runoff of sediments and nutrients from different land use types to coastal waters?* - *How and in what locations could the protection or restoration of forests and/or wetlands, the management of agricultural areas and/or roads, or the installation of land-based pollution controls be focused to minimize runoff to coastal waters?* - *How will any such pollution control installations have to be designed (including size, structural characteristics) and located to both accommodate projected sediment or nutrient runoff loads and also withstand the direct physical climate change impacts of larger, more intense storms, greater erosion, etc.?* |
| 1. Minimize nutrients and bacterial pollution from wastewater systems including sewage and septic systems | - *How will climate change-related shifts in precipitation patterns (e.g. storm frequency and intensity) affect the volume of water that must be handled by wastewater systems?* - *How will wastewater and septic systems need to be designed and located to accommodate projected increased rainfall, extreme events and sea level rise?* |
| 1. Minimize marine-based pollution due to solid waste, sewage, plastics, derelict fishing gear, and releases of hazardous materials from oil and gas installations, cruise ships, and shipping, etc. [2, 3, 12] | - *How will changes in the intensity and frequency of large storms affect the frequency, severity and location of spills, groundings, derelict fishing gear and other pollution inputs from marine-based installations and activities?* - *How can safety requirements, installation and equipment design, timing and location of activities, etc., be adjusted to counteract these effects?* |
| 1. Prevent direct habitat destruction from anchoring, ship groundings, and destructive fishing activities [6, 8, 11, 13] | - *How will climate change effects (e.g. changes in currents and tidal patterns) or changes in water depths from sea level rise, affect recreational and commercial boating and shipping patterns and fishing activities?* - *What are the cumulative impacts of climate change and destructive activities in marine managed areas?* - *Given this information, how should regulations or spatial and temporal restrictions be added or modified, and in what locations should enforcement be concentrated?* |
| 1. Prevent overfishing of coral reef-associated fish and invertebrate species, especially herbivores [5-7, 9, 14-19] | - *How will climate change effects on oceanographic conditions (e.g. increased sea surface temperature, changes in upwelling patterns from stratification of surface waters, changes in ocean circulation, and ocean acidification) directly affect the distribution, abundance, and recruitment patterns of fish and invertebrate populations, especially herbivorous species needed to manage algal overgrowth?* - *Will these or other impacts of climate change (e.g., increased runoff from more frequent/intense storms) also interact with other stressors (e.g., land-based pollutants) to further impact coral reef herbivores?* - *How would these climate change impacts interact with and/or exacerbate impacts from current levels of fishing effort and gear operation?* - *How can this information on the locations and extent of changes in fisheries production be factored into measures to reduce fishing effort and increase fish biomass on coral reefs?* |
| 1. Protect at least 20% (ideally 30%) of each habitat type within permanent no-take areas [8, 11, 13, 20] | - *How will climate change affect the extent, distribution, quality, and/or accessibility of various habitat types?* - *How should this information affect the selection of habitats (location, size, shape) as long-term no-take areas?* |
| 1. **Protect Key Ecosystem Features**   *Focus management on structural characteristics (e.g., geophysical stage), organisms, or areas (e.g., spawning sites) that represent important “underpinnings” or “keystones” of the current or future system of interest* | |
| 1. Protect special or unique sites [8, 11] | - *What is the vulnerability of special or unique sites to the direct effects of climate change?* - *Are there interactions between climate change and other stressors to special or unique sites that further affect their vulnerability?* - *Given this information, how do protections of these sites need to be adjusted in terms of prioritization among sites, stressors on which to focus, and design of management techniques?* |
| 1. Protect ecologically significant areas such as nursery grounds, spawning grounds, critical habitat for threatened and endangered species, and areas of high species diversity [11, 12, 21] | - *How will climate change affect the location and viability of ecologically significant areas (e.g. low lying sandy islets for sea turtle nesting; mangrove and seagrass areas as fisheries nursery grounds)?* - *How might practices and protections have to be adjusted or initiated to ensure the persistence of ecologically significant areas under climate change?* |
| 1. Manage functional species and groups necessary for maintaining the health of reefs and other ecosystems [7, 21-23] | - *What is the vulnerability of functional species and groups (e.g. herbivores, apex predators) to the interaction of climate change with other human-generated and natural stressors, and in what locations are they most vulnerable?* - *What management options can be employed, and in which locations, to minimize impacts on the most vulnerable species and groups?* |
| 1. Include linked ecological units in managed areas to help maintain ecosystem functions and resilience [11, 21, 24] | - *How will climate change affect supporting functions provided by coral reef-associated habitats* (e.g., adjacent mangroves and seagrasses)*?* - *What locations and which types of ecological units are most affected?* - *What are the implications of this information for selection of areas for protection and management?* |
| 1. Use temporal restrictions such as seasonal, closures and temporary zoning to protect spawning aggregations and manage fishing effort to support faster recovery during times of severe stress, such as a bleaching event or after extreme events. [8, 11, 12, 25] | - *How will climate-related events, such as El Niño, impact the seasonality and location of spawning aggregations?* - *How will timing of closures to protect spawning aggregations need to be adjusted to account for these effects, in order to support fisheries production and faster recovery after events?* |
| 1. Design managed areas with dynamic boundaries and buffers to protect breeding and foraging habits of highly migratory and pelagic species and allow shifting of species ranges [8, 11] | - *How will migratory and pelagic species’ ranges change in response to climate change, and what is the spatial variability of those changes?* - *How will climate change affect the condition, extent, and location of breeding and foraging habitats for those species?* - *Given this information, what should be the extent, location and configuration of boundaries and buffers to provide sufficient protection of breeding and foraging habitat, and how do those need to be adjusted over time to account for shifts in species ranges?* |
| 1. **Ensure Connectivity**   *Protect and restore habitats that facilitate movement of organisms (and gene flow) among resource patches* | |
| 1. Identify and manage networks of resilient reefs connected by currents [8, 11, 18, 23, 26] | - *Which areas have historically demonstrated resistance to/or recovery from exposure to climate change impacts?* - *Which areas are projected to have less exposure to climate change impacts (e.g. increased sea surface temperatures, decreased ocean pH) and could therefore serve as refugia?* - *How will climate change affect currents that provide connectivity between resilient areas?* - *Which areas have demonstrated resistance and/or recovery to climate change impacts?* - *What are the implications of this information for design of managed area networks to maximize connectivity and maintain it into the future?* |
| 1. Include a mixture of habitats and habitat edges (e.g., mangroves, seagrass) within systems of managed areas connected by currents [8, 9, 11, 27] | - *How will climate change affect adjacent habitats that are critical for various life history stages of reef species? ,* - *Which areas are most likely to retain high heterogeneity (e.g. rugosity, microhabitats) that may contribute to increased resilience to climate change?* - *How will projected changes in currents affect connectivity among these areas important for larval dispersal? What are the implications of this information for design of managed area networks?* |
| 1. Apply minimum size and include a variety of shapes to systems of managed areas [8, 11] | - *How will climate change affect currents that provide ecological connectivity among areas being considered for inclusion in a managed area system?* - *What are the implications of changes in current patterns for the optimal shape and size of the areas selected?* |
| 1. Consider managing areas upstream that can serve as “source” areas for larvae within the dominant current flow to improve downstream habitats and populations [8, 11] | - *How will projected changes in surface circulation affect ecological connectivity?* - *What are the implications of this information for identification of areas of the marine environment that support source populations of key species?* |
| 1. Separate no-take areas by 1 to 20 km apart (with a mode of ~1 to 10 km)[8] | - *How will projected changes in surface circulation affect ecological connectivity among no-take areas?* - *What are the implications of this information for determination of the maximum distance among no-take areas?* |
| 1. Identify ecological connections and apply that information to protect source populations in resistant areas that can provide recruitment and support recovery in other areas [6-9, 18, 21, 23, 28-31] | - *How will projected changes in surface circulation affect ecological connections among ecosystems?* - *What habitat characteristics, or exchanges among systems, contribute to resistance to climate change within the managed area and how might those change?* - *What implications does this information have for determining which source populations and areas that are resistant to climate impacts are priority for protection and management?* |
| 1. **Restore Structure and Function**   *Rebuild, modify or transform ecosystems that have been lost or compromised, in order to restore desired structures (e.g., habitat complexity) and functions (e.g., nutrient cycling)* | |
| 1. Enhance natural recovery processes following extreme events through active restoration [32, 33] | - *How will climate change affect the timing, frequency and intensity of extreme events (e.g. prolonged, high sea surface temperatures and damage from storm events)?* - *What species, habitats and locations will be most affected and least affected by extreme events, and what are the projected times of recovery for the most and least affected after the different types of events?* - *What are the implications of this information for targeting and designing restoration activities to maximize recovery following extreme events?* |
| 1. Re-establish source populations of corals with the highest sensitivity and lowest adaptive capacity to ecologically suitable areas with the lowest exposure to non-climate stressors and climate impacts [34-36] | - *How will the impacts of climate change vary for different species due to differences in sensitivity, exposure, and adaptive capacity?* - *What areas that currently have suitable habitat will remain so in the future, or are currently on the margin of habitat suitability (e.g., due to temperature regime) that could become more suitable after restoration activities because of low exposure to non-climate stressors and climate impacts?* - *Given the above information, which populations are vulnerable and should be reestablished, when, and in what locations to ensure their viability under climate change?* |
| 1. Restore herbivore populations of fish and invertebrates to increase resilience to coral bleaching and disease [12, 13, 16, 18, 22, 37-39] | - *How will climate change affect the growth and recruitment of important reef herbivores?* - *Which areas important to herbivores have demonstrated resistance and/or recovery to climate change impacts?* - *Given the different functional roles of reef herbivores and the different exposures reefs will face, what species or functional groups and locations should be the focus of management efforts?* |
| 1. Prevent and manage biofouling and invasive species to preserve the integrity of marine communities in managed areas [12, 20, 40] | - *How will climate-related changes in ocean currents, nutrient runoff, marine debris, and other factors affect the transport and growth of non-indigenous species?* - *What areas will be the biggest sources of non-indigenous species and what areas down-current are most important to protect?* - *Given the information above, which areas should be targeted for protection from or removal of non-indigenous species?* |
| 1. Control outbreaks of coral predators [12] | - *How will climate change affect populations of coral predators (e.g. crown-of-thorns starfish, encrusting and boring sponges, and Drupella snails?* - *What coral species and reefs will be most affected by outbreaks of these predators under climate change?* - *Given the information above, what predators should be targeted for control and in what areas?* |
| 1. Restore mangroves for multiple benefits including expansion of nursery habitat, improved water quality, release of tannins and other dissolved organic compounds that may reduce photo-oxidative stress in corals and carbon sequestration (“blue carbon”) [21, 24, 40, 41] | - *How will climate change, in combination with land use change, affect the extent of mangrove habitat and environmental conditions needed for successful mangrove restoration?* - *At what locations will conditions most favor successful mangrove restoration?* - *What are the implications of this information for selection and prioritization of sites for mangrove restoration, including where land use policies could be put in place to allow landward migration of mangroves in response to sea level rise?* |
| 1. Develop artificial reef complexity using electrochemical or geochemical modification of seawater or other methods [38, 42] | - *How will the effects of ocean acidification on coral skeleton growth vary among species and locations?* - *What are the implications of this information for placement and engineering design (size, configuration, strength of current) of electrochemical or geochemical modifications?* |
| 1. **Protect Refugia**   *Protect areas less affected by climate change as sources of “seed” for recovery (in the present) or as destinations for climate-sensitive migrants (in the future)* | |
| 1. Identify and protect current and future areas that appear to be resistant to climate change effects or that recover from climate-induced disturbances [6, 11, 12, 15, 16, 21, 23, 43, 44] | - *What areas will be less exposed or sensitive to climate impacts, or will recover quickly from a disturbance once it occurs, (e.g. areas with structurally complex reefs in deeper water, reefs with high density of juvenile corals and/or herbivorous fishes, reefs with low nutrient and/or sediment loads,, areas with localized upwelling or other cool-water conditions that protects corals from bleaching during large-scale temperature anomalies, areas with heat-resistant coral species, areas with cooler water runoff from land, areas where reef complexes are well flushed by oceanic water, areas with carbonate rich environments including raised limestone reefs and islands, extensive reef flats, patch reef/coral head complexes, reefs near carbonate sediment deposits)?* - *How will climate change affect the location of current and future refugia (e.g. changes in upwelling, circulation and residence time of water in reef ecosystems) that can be targeted for protection and use as destinations for future recruitment and/or relocation of coral reef species? Are there risk tradeoffs (e.g. areas with upwelling may see less heat stress, but can sometimes see cold-related die-offs)?* |
| 1. Establish dynamic managed areas with flexible boundaries that can be defined by large-scale oceanographic features such as oceanic fronts where changes in types and abundances of organisms often occur [21] | - *How will climate change effects (e.g. increasing sea surface temperature, sea level rise, and storm events) spatially and temporally affect the dynamics of large-scale oceanographic features and what is the variability around those spatial and temporal alterations?* - *How will this information alter the way these shifting managed areas should be defined or sited?* - *Given this information, what type and degree of flexibility in management strategies will be necessary (spatially and temporally) in order to effectively manage areas as oceanic features shift?* |
| 1. Protect habitats similar to those that thrived during the middle Holocene, when coral reefs flourished closer to the poles, to allow for range migration [40, 45] | - *What are the characteristics of the types of habitats that existed during the middle Holocene, and where are habitats with those characteristics projected to be in the future under climate change?* - *Given this information, what habitat types should be protected, and in which areas, as sites for range migrations?* |
| 1. Use artificial shading or water mixing when corals are exposed to thermal stress, to protect coral sites of specific importance from bleaching [40] | - *How is climate change expected to affect the location, magnitude and duration of temperature anomaly events with respect to coral sites of specific importance?* - *How can this information be used to select which reefs of specific importance might become particularly vulnerable to thermal stress in the future, and to determine when artificial shading or water mixing should be use, and what amount of light reduction or mixing will be needed to sufficiently counteract the interaction of temperature and UVR that induces bleaching?* |
| 1. Designate large managed areas in ways that increase the likelihood that they will contain some areas of natural refugia that escape temperature anomalies [8, 46] | - *What are the locations and scales of areas with less exposure to climate impacts now or in the future, (e.g., currents or other features that protect areas from temperature anomalies or a large managed area that contains all anticipated locations for poleward shift from the typical range)?* - *Given this information, what large/complex coral reefs should be protected and how large should managed areas be, and at what locations, to maximize the likelihood that they will contain some areas of natural refugia?* |
| 1. Identify and protect seagrass beds to provide a short-term local pH buffering effect for adjacent coral reefs [1] | - *How will climate change impact seagrass beds adjacent to coral reefs?* - *What are the locations and scales of seagrass habitat that can provide buffering capacity to adjacent reefs and calcifying organisms?* |
| 1. **Relocate Organisms**   *Engage in human-facilitated transplanting of organisms from nurseries or from one location to another in order to by-pass a barrier (e.g. environmental, genetic)* | |
| 1. Relocate corals to overcome environmental barriers or negative chemical cues to recruitment [47-56] | - *How will climate change contribute to environmental conditions (negative chemical cues from degraded reefs, disruption of connecting currents) that restrict recruitment from source reefs?* - *What are the implications of this information for selection of source and sink reefs for transplantation of coral fragments?* |
| 1. Establish coral nurseries and fish hatcheries for outplanting and restocking of species whose recruitment is negatively affected by climate change [34, 35, 42] | - *How will climate change contribute to environmental conditions (negative chemical cues from degraded reefs, disruption of connecting currents) that restrict recruitment from source reefs?* - *For which corals and fish will this pose the greatest problem by impeding recruitment to historically-connected sites?* - *Which of these organisms can be grown/hatched in nurseries for use in “assisted migration” to impeded sites?* |
| 1. **Support Evolutionary Potential**   *Protect a variety of species, populations and ecosystems in multiple places to bet-hedge against losses from climate disturbances, and where possible manage these systems to assist positive evolutionary change* | |
| 1. Protect species and populations with genetic, physiological, or ecological traits characteristic of low sensitivity and high adaptive capacity to climate impacts [23, 36, 57, 58] | - *How is climate change expected to affect the frequency, duration and magnitude of temperature anomaly events and other climate-related stressors?* - *Which species, assemblages or communities are likely to be most resistant to the negative effects of climate stressors and/or have greater recovery capacities after events?* - *Are the same species, assemblages, or communities resistant or resilient to different stressors (e.g. to both heat and acidification)?* - *What are the implications of this information for selection of sites for special protection?* |
| 1. Create “designer” reefs using human-assisted evolution with species that have beneficial genetic variants for and/or are acclimatized to higher sea surface temperatures and ocean acidification [50-56, 59, 60] | - *Which symbionts are most stress-tolerant?* - *Which coral species have the greatest genetic resistance and/or greatest ability to acclimatize to climate impacts?* - *How can this information be used to create and/or select species for special protections or for use in restoration of degraded reefs?* |
| 1. Protect species, population and genetic diversity within and across habitats [8, 11] | - *What are the factors that influence species, population and genetic diversity within different habitats, and how will climate change affect those factors?* - *Based on this information, how can protections within different habitats be designed to maximize characteristics that promote the greatest possible diversity?* - *What about selection of different managed areas within the broader landscape?* |
| 1. Replicate habitat types in multiple managed areas to spread risks associated with climate change [9, 21] | - *How will climate change affect the spatial scale and pattern of temperature anomaly events and other extreme events (and conversely, refugia)?* - *What about acidification or other stressors?* - *How can this information be used to select a suite of managed areas that best spread risks across space and time while maintaining genetic, population, and species diversity?* |
| 1. Ensure that the full breadth of habitat types is managed [21] | - *How do different habitat types (e.g., fringing reef, fore reef, back reef, patch reef) vary in their vulnerability to climate change across space and time?* - *Do areas with low vulnerability for one habitat type tend to have low vulnerability for other habitat types, or is there no relationship?* - *What are the implications of this information for how the spatial pattern of protection may have to be adjusted to ensure that the full breadth of habitats is protected?* |
| 1. Maximize habitat heterogeneity within managed areas and consider protecting large areas to preserve biodiversity, connectivity, ecological processes and functions and evolutionary potential [21] | - *How will climate change affect the spatial scale and pattern of temperature anomaly events and other extreme events (and conversely, refugia)? What about acidification or other stressors?* - *What are the scales and locations of areas with high habitat heterogeneity and biodiversity, and which of these areas are expected to be the least sensitive or exposed to climate change, or have high adaptive capacity?* - *What are the implications of this information for the design and size of managed areas to maintain habitat heterogeneity, connectivity and ecological processes and functions into the future?* - *Are there tradeoffs between selecting for areas where past exposure to climate stress has lead to lower sensitivity to future climate stress and areas with lower past exposure to climate stress but potentially higher sensitivity?* |

1. West, J.M. and S.H. Julius, *The Art of the Possible: Identifying Adaptation Options*, in *Climate-Smart Conservation: Putting Adaptation Principles into Practice*, B.A. Stein, et al., Editors. 2014, National Wildlife Federation: Washington, DC.

2. Burke, L., et al., *Reefs at Risk Revisited in the Coral Triangle*. 2012, World Resources Institute: Washington, DC. p. 72.

3. Burke, L., et al., *Reefs at Risk Revisited*. 2011, World Resources Institute: Washington, D.C. p. 114.

4. Maina, J., et al., *Human deforestation outweighs future climate change impacts of sedimentation on coral reefs.* Nature Communications, 2013. **4**: p. 1-7.

5. Kennedy, E.V., et al., *Avoiding coral reef functional collapse requires local and global action.* Current Biology, 2013. **23**: p. 912–918.

6. Maynard, J., et al., *Building resilience into practical conservation: identifying local management responses to global climate change in the southern Great Barrier Reef.* Coral Reefs, 2010.

7. Flower, K.R., et al., *Toward Ecosystem-based Coastal Area and Fisheries Management in the Coral Triangle: Integrated Strategies and Guidance*. 2013, Coral Triangle Initiative Support Program for the U.S. Agency for International Development: Jakarta, Indonesia.

8. Fernandes, L., et al., *Biophysical principles for designing resilient networks of marine protected areas to integrate fisheries, biodiversity and climate change objectives in the Coral Triangle. Report prepared by The Nature Conservancy for the Coral Triangle Support Partnership.* 2012: p. 152.

9. McLeod, E., et al., *Preparing to manage coral reefs for ocean acidification: lessons from coral bleaching.* Frontiers in Ecology and the Environment, 2012. **11**(1): p. 20-27.

10. D’Angelo, C. and J. Wiedenmann, *Impacts of nutrient enrichment on coral reefs: new perspectives and implications for coastal management and reef survival.* Current Opinion in Environmental Sustainability, 2014. **7**: p. 82-93.

11. Green, A., et al., *Designing marine reserves for fisheries management, biodiversity conservation and climate change adaptation.* Coastal Management, 2014. **42**: p. 143-159.

12. The Nature Conservancy. *Reef Resilience: Coral Reef Module* 2015; Available from: <http://www.reefresilience.org/coral-reefs/management-strategies/>.

13. Lamb, J.B., et al., *Protected areas mitigate diseases of reef-building corals by reducing damage from fishing.* Ecology, 2015.

14. McLeod, E., et al., *Integrating reef resilience and climate change vulnerability into protected area design and management in Palau and greater Micronesia. A Report Prepared for the Western Pacific Coral Reef Institute, University of Guam. Prepared by TNC, Palau Internation Coral Reef Center, and WWF*. 2012. p. 75.

15. McClanahan, T.R., et al., *Prioritizing Key Resilience Indicators to Support Coral Reef Management in a Changing Climate.* PLoS ONE, 2012. **7**(8): p. e42884.

16. Edwards, H.J., et al., *How much time can herbivore protection buy for coral reefs under realistic regimes of hurricanes and coral bleaching?* Global Change Biology, 2010.

17. Anthony, K.R.N., et al., *Ocean acidification and warming will lower coral reef resilience.* Global Change Biology, 2011. **17**(5): p. 1798-1808.

18. Dodge, R.E., et al., *A Call to Action for Coral Reefs.* Science, 2008. **322**: p. 189-190.

19. MacNeil, M.A., et al., *Recovery potential of the world's coral reef fishes.* Nature, 2015. **advance online publication**.

20. Convention on Biological Diversity, *Quick guides to the: Aichi Biodiversity Targets. Version 2 - February 2013*. 2013.

21. Keller, B.D., et al., *Climate Change, Coral Reef Ecosystems, and Management Options for Marine Protected Areas.* Environmental Management, 2009.

22. Cheal, A.J., et al., *Spatial variation in the functional characteristics of herbivorous fish communities and the resilience of coral reefs.* Ecological Applications, 2013. **23**(1): p. 174–188.

23. West, J.M. and R.V. Salm, *Resistance and resilience to coral bleaching: implications for coral reef conservation and management.* Conservation Biology, 2003. **17**(4): p. 956-967.

24. Zarate-Barrera, T.G. and J.H. Maldonado, *Valuing blue carbon: carbon sequestration benefits provided by the marine protected areas in Colombia.* PLoS One, 2015. **10**(5): p. e0126627.

25. Cinner, J.E., et al., *Periodic Closures as Adaptive Coral Reef Management in the Indo-Pacific.* Ecology and Society, 2006. **11**(1).

26. Olds, A.D., et al., *Mangrove-reef connectivity promotes the effectiveness of marine reserves across the western Pacific.* Global Ecology and Biogeography, 2013.

27. Olds, A.D., et al., *Synergistic effects of reserves and connectivity on ecological resilience.* Journal of Applied Ecology, 2012. **49**(6): p. 1195-1203.

28. Munday, P., et al., *Climate change and coral reef connectivity.* Coral Reefs, 2009. **28**(2): p. 379-395.

29. Cowen, R.K. and S. Sponaugle, *Larval dispersal and marine population connectivity.* Annual Review of Marine Science, 2009. **1**(1): p. 443-466.

30. Cowen, R.K., et al., *Population connectivity in marine systems: an overview.* Oceanography, 2007. **20**(3): p. 14-21.

31. Green, A., et al., *Larval dispersal and movement patterns of coral reef fishes, and implications for marine reserve network design.* Biological Reviews, 2014: p. n/a-n/a.

32. Ogston, A.S. and M.E. Field, *Predictions of Turbidity Due to Enhanced Sediment Resuspension Resulting from Sea-Level Rise on a Fringing Coral Reef: Evidence from Molokai, Hawaii.* Journal of Coastal Research, 2010. **26**(6): p. 1027–1037.

33. Ferrario, F., et al., *The effectiveness of coral reefs for coastal hazard risk reduction and adaptation.* Nature Communications, 2014. **5**.

34. Selig, E.R. and J.F. Bruno, *A Global Analysis of the Effectiveness of Marine Protected Areas in Preventing Coral Loss.* PLoS ONE, 2010. **5**(2): p. e9278.

35. Griffin, S., et al. *Scaling up Acropora nurseries in the Caribbean and improving techniques*. in *Proceedings of the 12th International Coral Reef Symposium. 9-13 July 2012 Cairns, Queensland, Australia. ARC Centre of Excellence for Coral Reef Studies, James Cook University, Townsville, Queensland, Australia*. 2012.

36. Foden, W.B., et al., *Identifying the World's Most Climate Change Vulnerable Species: A Systematic Trait-Based Assessment of all Birds, Amphibians and Corals.* PLoS ONE, 2013. **8**(6): p. e65427.

37. Maynard, J., et al., *Projections of climate conditions that increase coral disease susceptibility and pathogen abundance and virulence.* Nature Climate Change, 2015. **advance online publication**.

38. Rogers, A., et al., *Anticipative management for coral reef ecosystem services in the 21st century.* Global Change Biology, 2015. **21**(2): p. 504-514.

39. Streit, R., A. Hoey, and D. Bellwood, *Feeding characteristics reveal functional distinctions among browsing herbivorous fishes on coral reefs.* Coral Reefs, 2015: p. 1-11.

40. Hollarsmith, J.A., S.P. Griffin, and T.D. Moore. *Success of outplanted Acropora cervicornis colonies in reef restoration*. in *Proceedings of the 12th International Coral Reef Symposium. 9-13 July 2012 Cairns, Queensland, Australia. ARC Centre of Excellence for Coral Reef Studies, James Cook University, Townsville, Queensland, Australia*. 2012.

41. McLeod, E. and R.V. Salm, *Managing Mangroves for Resilience to Climate Change*. 2006, IUCN: Gland, Switzerland. p. 64.

42. Rau, G.H., E. McLeod, and O. Hoegh-Guldberg, *The need for new ocean conservation strategies in a high-carbon dioxide world.* Nature Climate Change, 2012. **2**(10): p. 720-724.

43. Guest, J.R., et al., *Contrasting Patterns of Coral Bleaching Susceptibility in 2010 Suggest an Adaptive Response to Thermal Stress.* PLoS ONE, 2012. **7**(3): p. e33353.

44. Graham, N.A.J., et al., *Predicting climate-driven regime shifts versus rebound potential in coral reefs.* Nature, 2015. **518**(7537): p. 94-97.

45. Baird, A.H., B. Sommer, and J.S. Madin, *Pole-ward range expansion of Acropora spp. along the east coast of Australia.* Coral Reefs, 2012. **31**: p. 1063.

46. Selig, E.R., K.S. Casey, and J.F. Bruno, *Temperature-driven coral decline: the role of marine protected areas.* Global Change Biology, 2012. **18**: p. 1561–1570.

47. D'Angelo, C., et al., *Local adaptation constrains the distribution potential of heat-tolerant Symbiodinium from the Persian/Arabian Gulf.* ISME Journal, 2015: p. 1-10.

48. Dixson, D.L., D. Abrego, and M.E. Hay, *Chemically mediated behavior of recruiting corals and fishes: A tipping point that may limit reef recovery.* Science, 2014. **345**(6199): p. 892-897.

49. Bruno, J.F., *How do coral reefs recover?* Science, 2014. **345**(6199): p. 879-880.

50. Mascarelli, A., *Designer Reefs: Biologists are directing the evolution of corals to prepare them to fight climate change.* Nature, 2014. **508**: p. 444-446.

51. Seneca, F.O. and S.R. Palumbi, *The role of transcriptome resilience in resistance of corals to bleaching.* Mol Ecol, 2015. **24**(7): p. 1467-84.

52. Bay, R.A. and S.R. Palumbi, *Multilocus adaptation associated with heat resistance in reef-building corals.* Current Biology, 2014. **24**: p. 1-5.

53. Barshis, D.J., et al., *Genomic basis for coral resilience to climate change.* Proceedings of the National Academy of Sciences, 2013. **110**(4): p. 1387-1392.

54. Logan, C.A., et al., *Incorporating adaptive responses into future projections of coral bleaching.* Global Change Biology, 2014. **20**(1): p. 125-139.

55. Palumbi, S.R., et al., *Mechanisms of reef coral resistance to future climate change.* Science, 2014. **344**(6186): p. 895-898.

56. Voolstra, C.R., et al., *Rapid evolution of coral proteins responsible for interaction with the environment.* PLoS One, 2011. **6**(5): p. e20392.

57. Darling, E.S., T.R. McClanahan, and I.M. Côté, *Life histories predict coral community disassembly under multiple stressors.* Global Change Biology, 2013. **19**: p. 1930-1940.

58. Noreen, A.M.E., et al., *Diverse associations among coral host haplotypes and algal endosymbionts may drive adaptation at geographically peripheral and ecologically marginal locations.* Journal of Biogeography, 2015. **42**(9): p. 1639-1650.

59. van Oppen, M.J.H., et al., *Building coral reef resilience through assisted evolution.* Proceedings of the National Academy of Sciences, 2015. **112**(8): p. 2307-2313.

60. Putnam, H.M. and R.D. Gates, *Preconditioning in the reef-building coral Pocillopora damicornis and the potential for trans-generational acclimatization in coral larvae under future climate change conditions.* Journal of Experimental Biology, 2015. **218**(15): p. 2365-2372.

1. The Compendium maintains the seven general adaptation strategies from West & Julius (2014) as a relevant and useful framework for identifying adaptation options for coral reef ecosystems. The example adaptation options and climate-smart design considerations in the Compendium are meant to be illustrative rather than comprehensive and to stimulate thinking of site-relevant possibilities. As new research and practices emerge, the range of examples will continue to grow and the Compendium will need to be reviewed and updated over time. [↑](#footnote-ref-2)
